# Prior vaccinations improve immunogenicity of inactivated influenza vaccine in young children aged 6 months to 3 years: A cohort study

**DOI:** 10.1097/MD.0000000000011551

**Published:** 2018-07-20

**Authors:** Kazuya Ito, Ayumi Mugitani, Shin Irie, Motoki Ishibashi, Yoshio Takasaki, Shizuo Shindo, Takashi Yokoyama, Yuji Yamashita, Keigo Shibao, Hideki Koyanagi, Wakaba Fukushima, Satoko Ohfuji, Akiko Maeda, Tetsuo Kase, Yoshio Hirota

**Affiliations:** a Department of Public Health, Osaka City University Graduate School of Medicine; Research Center for Infectious Disease Sciences, Osaka City University Graduate School of Medicine, Osaka; b SOUSEIKAI Clinical Epidemiology Research Center; c College of Healthcare Management, Fukuoka; d SOUSEIKAI Sumida Hospital, Tokyo; e SOUSEIKAI; f SOUSEIKAI, PS Clinic; g Takasaki Pediatric Clinic; h Shindo Children's Clinic; i Yokoyama Pediatric Clinic; j Yamashita Pediatric Clinic; k Shibao Clinic, Fukuoka; l SOUSEIKAI Dodo Clinic, Tokyo, Japan.

**Keywords:** immunogenicity, inactivated influenza vaccine, prior vaccination, seroprotection rate, young children

## Abstract

In young children, infrequent antigen exposure, which is partly characterized by fewer vaccinations, may be a factor impairing the immunogenicity of inactivated influenza vaccine.

We assessed the effects of prior vaccinations on age-specific immune responses in Japanese children aged 6 months to 3 years, using data from a cohort study with 266 children who had received 2 doses (0.25 mL/dose for < 3 years old, 0.5 mL/dose for 3 years old) in the 2006/2007 season. Serological measures, primarily seroprotection rates, between previously vaccinated and vaccine-naïve children were compared within 1-year age strata. The seroprotection rate was defined in 2 ways as the proportion of subjects who achieved an antibody titer of 1:40 or 1:160. Multivariate logistic regression was also performed to estimate the independent effect of prior vaccination on seroprotection rate.

After the first dose, seroprotection rates with the threshold of 1:40 in vaccine-naïve 1-year-olds remained low (28% for AH1, 26% for AH3, 2% for B), similar to those of 0-year-olds. In contrast, seroprotection rates in previously vaccinated 1-year-olds (77% for AH1, 86% for AH3, 18% for B) were significantly higher than those in vaccine-naïve 1-year-olds. These seroprotection rates for AH1 and AH3 were comparable with those in previously vaccinated 2- and 3-year-olds. Although seroprotection rates for B remained low in every age stratum even after the second dose, seroprotection rate in previously vaccinated 1-year-olds (50%) was similar to that in 3-year-olds. After adjustment for age, baseline antibody titer and experience of acute febrile respiratory illness in the preceding season, odds ratios showed a significant independent positive effect of prior vaccination on seroprotection rate for every strain. After the seroprotection threshold was changed from 1:40 to 1:160, the results of the effects of prior vaccinations on immunogenicity were similar or became more evident, which demonstrate the robustness of our findings.

Our study found that prior vaccinations improved poor immunogenicity among young children, especially in 1-year-olds.

## Abstract 2

Supplemental Digital Content is available in the text

## 1 Introduction

The intrinsic immaturity of the immune system in young children and infrequent antigen exposure may impair their immune response to inactivated influenza vaccine (IIV). The World Health Organization and the United States Advisory Committee on Immunization Practices recommended 2 doses of IIV to induce a sufficient level of antibodies for children aged < 9 years in their first season of vaccination.^[1,2]^ A study of vaccine-naïve children aged 6 to 23 months in the United States observed that, although younger children showed poorer immunogenicity, antibody titers achieved a protective level after 2 doses in a high proportion (>80%) of children for almost every strain, even in the youngest children aged 6 to 11 months.^[3]^ However, few other studies have assessed age-specific immunogenicity.^[4,5]^ Little is known about the effect of prior vaccinations on immunogenicity in children aged ≤ 3 years, although a number of studies have addressed the effects of prior vaccinations on immunogenicity in older children or adults and reported that prior vaccinations reduced the immune responses to IIV.^[6–9]^

Here, we assessed the age-specific immunogenicity of IIV and the effects of prior vaccinations on immunogenicity across the age of 6 months to 3 years by 1-year age strata, using data from a cohort study on the immunogenicity of IIV conducted in the 2006/2007 season in Japan. In that study, the dosage of IIV was the same as the current standard, namely 0.25 mL/dose for children < 3 years and 0.5 mL/dose for those aged 3 years.

## 2 Materials and methods

### 2.1 Study subjects

The study subjects were children aged 6 months to 3 years recruited from 6 pediatric practices in Fukuoka and Tokyo, Japan. We attempted to register approximately 50 children in each 1-year age stratum (0-, 1-, 2-, and 3-year-olds) from October to December in 2006. Children were excluded from the study if they had an acute febrile illness at the time of vaccination, a history of anaphylaxis to vaccine components, or other conditions that precluded them from receiving the vaccine. The study protocol was approved by the ethics committee of Osaka City University Graduate School of Medicine and by the institutional review board of SOUSEIKAI. The guardians of all subjects provided written informed consent. The study was registered in the UMIN Clinical Trials Registry (UMIN000000494).

### 2.2 Vaccination

Vaccinations were performed using a single lot of a licensed trivalent inactivated, thimerosal-free, unadjuvanted influenza vaccine (FLUBIK HA, Lot HE05A, the Research Foundation for Microbial Diseases of Osaka University, Osaka, Japan) containing A/New Caledonia/20/99 (H1N1), A/Hiroshima/52/2005 (H3N2) and B/Malaysia/2506/2004. These vaccines included 30 μg/mL of hemagglutinin from each of the 3 strains. All subjects received 2 subcutaneous injections of IIV into their arms, 4 weeks apart, regardless of prior vaccination history. The dosage was 0.25 mL/dose for subjects < 3 years and 0.5 mL/dose for those aged 3 years.

At the time this study was carried out, the standard dosage in Japan had been set as 0.1 mL/dose for children aged 6 months to < 1 year and 0.2 mL/dose for children aged 1 to 5 years. However, the efficacy of such small-dose vaccination was disputed. The present study was undertaken to provide evidence regarding the immunogenicity of a higher dosage of IIV, namely 0.25 mL/dose for children aged 6 months to < 3 years and 0.5 mL/dose for those aged 3 years, which have been the standard in Japan since 2011. This means that the “previously vaccinated” subjects had received a lower dosage than the current standard.

### 2.3 Information collection

At the time of recruitment, the following information was collected using a self-administered questionnaire which was completed by the guardians: age, sex, history of IIV vaccination during the prior 3 seasons, and experience of acute febrile respiratory illness (FRI) of ≥ 39°C in the preceding season. Regarding the history of IIV, we did not collect information on the number of doses in each season. The pediatricians provided the following medical information in the preceding 6 months: treatment with steroids or other immunosuppressants, use of aspirin, acute illness, and underlying illness. In addition, we also prospectively surveyed the health conditions that would relate to influenza from enrollment to April 30, 2007, which was generally expected to be the end of the influenza season in Japan, using a self-report diary for the guardians: body temperature, respiratory symptoms, visit to a medical facility, result of a rapid diagnostic test and physician's diagnosis of influenza.

### 2.4 Measurement of antibody titer

Serum samples were collected from subjects at 3 time points: before vaccination (S0), 4 weeks after the first dose (S1) and 4 weeks after the second dose (S2). All serum samples were frozen-stored at –80°C to -70°C until simultaneously assayed at the Kannonji Institute, the Research Foundation for Microbial Diseases of Osaka University. Serum hemagglutination inhibition antibody titers for each vaccine antigen strain were measured by the standard method using type O human erythrocytes.

### 2.5 Statistical analysis

The subjects were stratified into 1-year age strata (0-, 1-, 2-, 3-year-olds). The following outcomes were calculated to assess the immunogenicity of IIV: geometric mean titer (GMT) and seroprotection rate. The seroprotection rate was defined as the proportion of subjects who achieved an antibody titer of 1:40 or 1:160 (SP^40^, SP^160^). A serum hemagglutination inhibition antibody titer of 1:40 has been reported to reduce the risk of contracting influenza by 50% in adults. Recently, a study has suggested that an antibody titer of 1:110 is a more relevant threshold of seroprotection as it correlates with a 50% risk reduction in children.^[10]^ Therefore, we set 1:160 as the secondary threshold of seroprotection, as it is the nearest value to 1:110 in our scale of hemagglutination inhibition titer measurement. The simple abbreviation of “SP” refers to both SP^40^ and SP^160^, unless otherwise specified. For analysis, titers <1:10 were regarded as 1:5, and reciprocal antibody titers were analyzed after logarithmic transformation. The results were presented in the original scale after calculating the antilogarithms. In a preliminary analysis, GMTs and SPs were compared among age strata using the Kruskal–Wallis rank test and Cochran–Armitage trend test, respectively. Within each 1-year age stratum, the effect of prior vaccination for SPs was assessed using the *χ*^*2*^ test. The exact test method was used as necessary. Subjects were categorized as “previously vaccinated” if they had received IIV in at least 1 of the prior seasons. The dose-response effect of the number of prior vaccinated seasons on SP was also assessed. Furthermore, the adjusted odds ratio (OR) and its 95% confidence interval of the prior vaccination for SP were estimated by a multivariate logistic model, which included age, baseline antibody titer level and experience of FRI in the preceding season as potential confounders. Because 0-year-olds were not able to have previous vaccinations, they were excluded from the multivariate analysis. One subject aged 2 years had missing data on the experience of FRI. For analysis, we assumed that the subject had no experience of FRI in the preceding season. All tests were 2-sided, and p-values < 0.05 were considered statistically significant. The calculations were performed with SAS ver. 9.3 (SAS Institute Inc., Cary, NC).

## 3 Results

**Figure 1:**
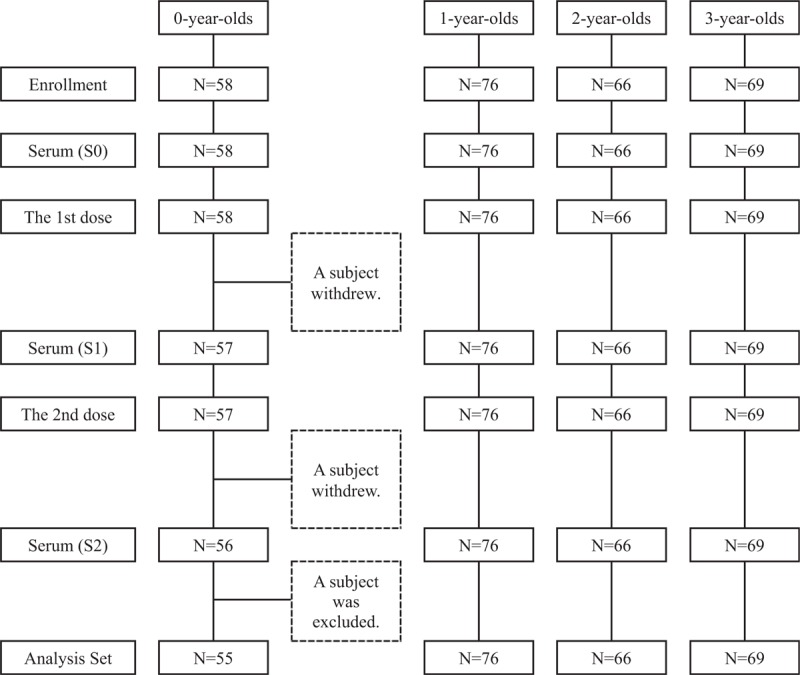
Selection of analysis set. S0: Serum samples before vaccination; S1: Serum samples 4 weeks after the first dose; S2: Serum samples 4 weeks after the second dose; N: Number of subjects. The withdrawals of 2 subjects have been independent of immunogenicity. All serum samples were simultaneously assayed. So, all study participants, including the pediatricians and guardians of subjects, were not notified of the antibody titers of their children during the serum-sampling period. A subject whose dosing interval was 2 weeks was excluded from the analysis set.

A total of 269 children were enrolled. No subject had received treatment with aspirin or immunosuppressive agents during the 6 months before enrollment. They received the first dose of IIV during October 5 to December 15, 2006. Following this, 268 received the second dose during November 1, 2006 to January 12, 2007. Triplicate sera data were available for 267 subjects. One subject whose dosing interval was 2 weeks was excluded from the analysis set. Finally, 266 subjects were included in the analysis (55 subjects were 0-year-olds, 76 were 1-year-olds, 66 were 2-year-olds and 69 were 3-year-olds) as shown in Figure 1. Our surveys showed that none of them had physician-diagnosed influenza during the serum-sampling period.

### 3.1 Baseline characteristics

Subject characteristics at recruitment are shown in Table 1. The proportion of subjects with prior vaccination increased with age (29% at age 1 year and 88% at 3). The proportion of subjects with experience of FRI in the preceding season ranged from 43% to 51% among 1- to 3-year-olds, whereas that in 0-year-olds was 11%. The proportion of subjects with a seronegative titer (< 1:10) was higher in the younger age strata than in the older age strata for every strain.

**Table 1:**
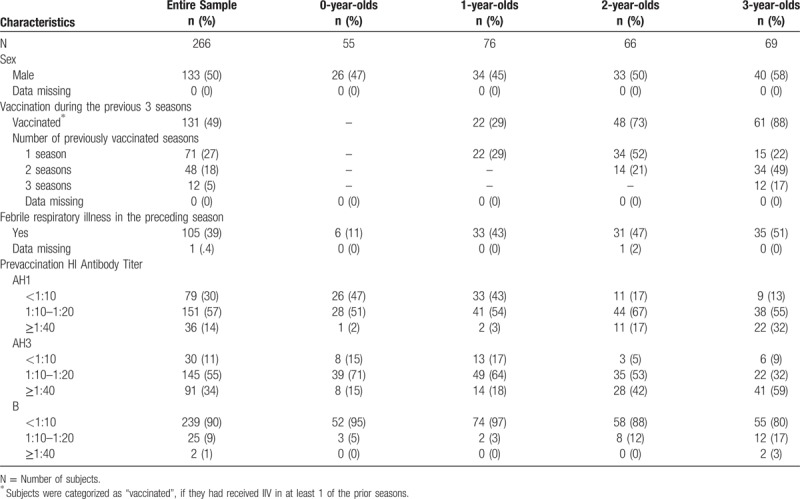
Baseline Characteristics.

### 3.2 Age-specific immune response

At every point of serological measurement (ie, S0, S1 and S2), both the GMTs and SP^40^s decreased significantly as age decreased for all 3 strains (Table 2). Especially for AH3, even after 2 doses, the GMT in 0-year-olds was less than 1:40, and the SP^40^s in 0- and 1-year-olds were 50% or less. With regard to B, the immune response was quite low regardless of age, with the highest GMT and SP^40^ for B of only 1:37 and 51%, respectively, after the second dose even in 3-year-olds.

**Table 2:**
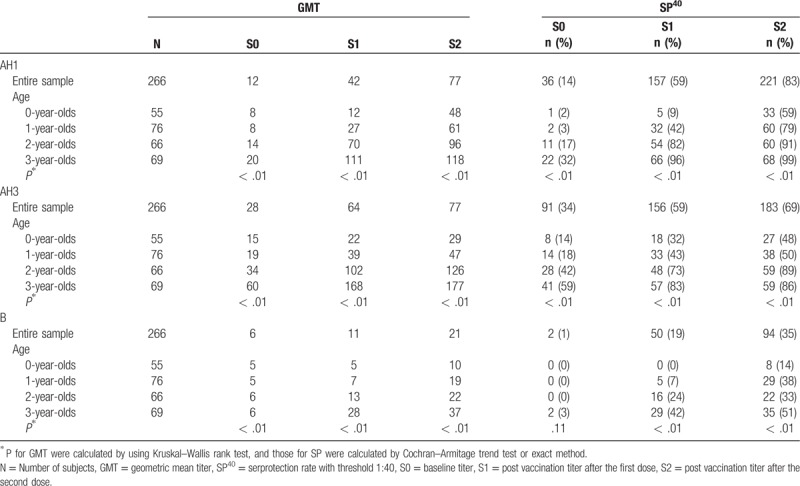
Summary of age-specific immune responses.

### 3.3 Association between prior vaccination and age-specific immune response

The SP^40^s in the previously vaccinated subjects were compared with those in the vaccine-naïve subjects within each 1-year age stratum (Table 3). The SP^40^s at baseline (S0) were not significantly different between the previously vaccinated and vaccine-naïve subjects within any age stratum regardless of strain. After the vaccines had been administered, the SP^40^s in the vaccine-naïve 1-year-olds remained poor for every strain, which were similar to those in 0-year-olds. For example, the SP^40^s for AH3 in the vaccine-naïve 1-year-olds and 0-year-olds were 26% versus 32% after the first dose and 31% versus 48% after the second, respectively. However, the SP^40^ after the first dose in the previously vaccinated 1-year-olds was significantly higher than that in the vaccine-naïve 1-year-olds for every strain (77% vs 28% for AH1, 86% vs 26% for AH3, 18% vs 2% for B). Overall, the SP^40^s for A strains in the previously vaccinated 1-year-olds were comparable to those in the previously vaccinated 2- and 3-year-olds. For B, the SP^40^ in the previously vaccinated 1-year-olds rose to a level (50%) similar to that of 3-year-olds after the second dose. In the 2- and 3-year-olds, the SP^40^s for AH1 in the vaccine-naïve subjects achieved a level similar to those of the previously vaccinated subjects.

**Table 3:**
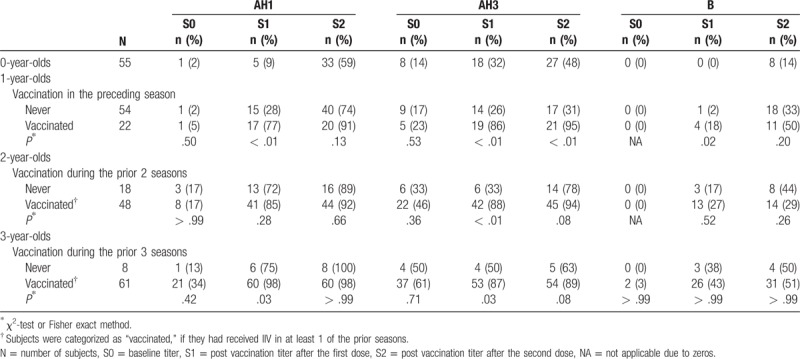
Seroprotection rate with a threshold of 1:40 according to prior vaccination within each age stratum.

Among the 2- and 3-year-olds who had received vaccines in at least 1 previous season (Supplementary Table 1), SP^40^s for A strains did not significantly differ across the number of prior vaccinated seasons. SP^40^s were sufficiently high even in the subjects who had been previously vaccinated in 1 season only (85%–100%). For B, only 3-year-olds who had received vaccines in all 3 seasons achieved high SP^40^s (75% after the first dose, 67% after the second dose), otherwise SP^40^s for B were ≤ 50% even after the second dose.

### 3.4 Independent association between prior vaccination and immune response

Multivariate analysis showed a significant association between prior vaccinations and SP^40^s after the first dose for every strain (multivariate OR: 5.2 for AH1, 26 for AH3 and 3.0 for B), independent of age, baseline antibody titer and experience of FRI in the preceding season (Table 4). With regard to the effects of covariates on SP^40^s, no relationship was observed between the SP^40^s and FRI in the preceding season. A higher baseline titer was positively associated with the SP^40^s, with significance. The older age showed a significant upward trend in multivariate ORs, except for AH3.

**Table 4:**
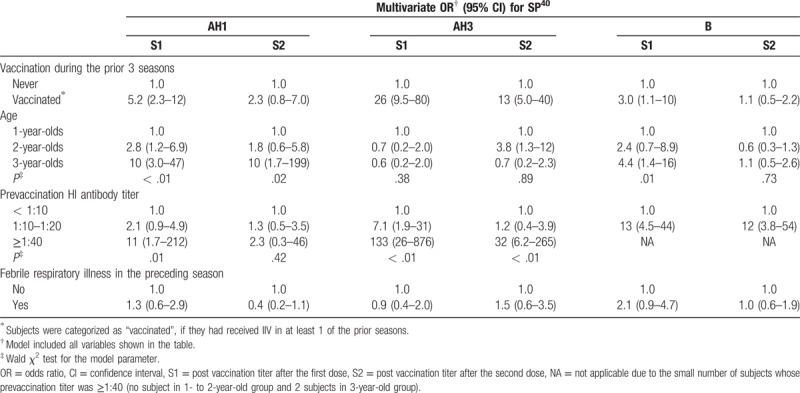
Effect estimates of prior vaccination on seroprotection rate with a threshold of 1:40.

To examine the influence of prior vaccination status on the association between age and SP^40^ for AH3, we removed the variable of prior vaccination from the multivariate model (Supplementary Table 2). For AH3, we clearly found greater multivariate ORs for the SP^40^ in older age groups with a significant upward trend, which were in consistent with the other 2 strains.

### 3.5 Robustness of results with a changing in threshold of seroprotection from 1:40 to 1:160

We assessed the previous vaccination effects on SP^160^ instead of SP^40^ in Tables 3 and 4. Using the same methodology, findings for the SP^160^s (Supplementary Table 3 and Supplementary Table 4) did not substantially differ from those for the SP^40^s.

Within age strata (Supplementary Table 3), the SP^160^s were lower than the SP^40^s for every strain regardless of age. However, the differences in SP^160^s for A strains between the previously vaccinated and vaccine-naïve subjects were still statistically significant, and became more evident compared with the differences in SP^40^, regardless of age, with the following 2 exceptions. First, in the 2-year-olds, the SP^160^ for AH1 in the previously vaccinated subjects was significantly lower than that in the vaccine-naïve subjects after the second dose. Second, in the 3-year-olds, the difference in SP^160^s for AH3 due to prior vaccination was smaller than the difference in SP^40^s after each dose. The SP^160^s for B strains were quite low, with a maximum value of only 21% after the second dose in the previously vaccinated 3-year-olds.

Multivariate analysis (Supplementary Table 4) detected significant independent effects of prior vaccinations on the SP^160^s after the first dose for A strains (multivariate OR: 7.1 for AH1, 19 for AH3). For B strain, the point estimate of multivariate OR (4.5) was almost the same as that for SP^40^ (3.0, see Table 4), albeit without significance. Additionally, experience of FRI in the preceding season significantly increased SP^160^ for AH3 (multivariate OR: 2.7 after the first dose; 2.8 after the second dose). Regarding the effects of age on SP^160^s, no relationship was observed for A strains. However, after prior vaccination status was removed from the model for SP^160^, point estimates of the multivariate ORs showed that SP^160^s tend to increase with increasing age, albeit without significance for AH3 (Supplementary Table 5).

## 4 Discussion

This study showed that prior vaccinations improved the immune response to IIV in children aged 1 to 3 years. The previously vaccinated 1-year-olds acquired a level of antibodies that was comparable to those of the previously vaccinated 2- and 3-year-olds. In contrast, the immune response in the vaccine-naïve 1-year-olds remained quite low, and similar to that of 0-year-olds. Since the SP^40^s at baseline did not significantly differ between the previously vaccinated and the vaccine-naïve subjects, the difference in SP^40^s after vaccination could be attributed to the prior vaccination effect. Multivariate analysis confirmed that the effect of prior vaccination on SP^40^ was significant and independent of age, baseline antibody titer and experience of FRI in the preceding season. To our knowledge, this is the first assessment of the change in immune response across the ages of 1 to 3 years. After the seroprotection threshold was changed from 1:40 to 1:160, the results of the effects of prior vaccinations on immunogenicity were similar or became more evident, which demonstrate the robustness of our findings.

It is generally perceived that the extent of the immune response in young children is low partly due to infrequent antigen exposure. In fact, one study suggested that a lack of prior infections in younger age children led to a low amount of influenza-specific memory B-cells and influenza-specific IgG-secreting cells in vaccine-naïve children aged 6 months to 4 years.^[11]^ In contrast, another study of vaccine-naïve children aged 2 to 3 years showed that children with prior infections acquired a higher amount of influenza-specific IgG-secreting cells after stimulation of IIV, compared with children without prior infection.^[12]^ With regard to our observations of the great increases in SPs in the prior vaccinated 1-year-olds, a substantial amount of influenza-specific memory B-cells might be induced by prior vaccinations and persist into the subsequent season even in infants. Young children may be able to compensate for the lack of prior infections with prior vaccinations in terms of the induction of substantial serum antibodies.

Compared with 1-year-olds, the differences in SPs due to prior vaccinations diminished in 2- or 3-year-olds. Baseline SPs were increased with age for every strain regardless of prior vaccination status. Similar phenomena have been observed in some studies of older children or adults, in which prior vaccinations reduced the additional increase in serum antibodies, influenza-specific memory B-cells, IgG-secreting cells or IFN gamma-secreting CD4^+^ T-cells after vaccination in the subsequent season.^[6–9]^ One study of adults aged 22 to 49 years proposed a possible immune mechanism in which preexisting antibodies could form antigen-antibody complexes with hemagglutinin protein of IIV injected into serum, so that it may reduce the amount of hemagglutinin protein available for stimulating naïve or memory B-cells.^[7]^ A recent study reconfirmed that the amount of preexisting serum antibodies negatively correlated with the response of influenza-specific memory B-cells to IIV among previously vaccinated children aged 3 to 17 years.^[13]^

Additionally, natural infections may also contribute to diminishing the differences in SPs due to prior vaccinations in our older subjects. Among our 2- and 3-year-olds, the effect of prior vaccinations on SP^40^s seemed to be reduced, particularly for AH1. In these ages, even if subjects were vaccine-naïve, > 70% of subjects acquired an antibody titer level of ≥ 1:40 for AH1 after the first dose. Also, SP^160^ for AH3 in the vaccine-naïve 3-year-olds achieved a level similar to that in the previously vaccinated 3-year-olds. According to the national surveillance system, both vaccine strains of AH1 and AH3 in the study season had been antigenically similar to the strains that circulated in the preceding season. The AH1 strain had circulated for at least the prior 3 seasons, and the AH3 strain was the predominant strain that circulated in the preceding season. Thus, it was possible that the subjects had been exposed to similar antigens through natural infection, even if the subjects were vaccine-naïve.

Taken together, these findings show that prior exposures to IIV may be necessary to induce a substantial immune response for IIV in the subsequent season especially in 1-year-olds, overwhelming their immune immaturity as a result of infrequent antigen exposure. Prior vaccinations play the role straightforwardly in 1-year-olds without modification by the multilayered profiles of several infections and vaccinations. The extent of the effect of prior vaccinations on improvement of immunogenicity may be reduced in 2- or 3-year-olds as exposure to natural infections or vaccinations increases. Further studies are needed to reveal the potential immune mechanism behind our findings in children aged 3 years and younger.

We found a favorable effect of prior vaccination on the immune response especially in 1-year-olds for every strain. Although the vaccine strain of AH3 had changed across the 4 seasons that were examined, but not AH1, the immune responses to AH3 were similar to or greater than those to AH1 in the previously vaccinated subjects in every age group. Our findings were consistent with those of a study that showed prior vaccination improved the immune response even when vaccine strain was changed in the subsequent season.^[14]^ Recently, 2 studies of the same subjects aged 3 to 17 years assessed vaccine or drifted antigen-specific immunities elicited by an IIV. As the results of the studies, the amount of drifted antigen-specific IgG-secreting cells was strongly correlated with that of vaccine antigen-specific IgG-secreting cells (correlation coefficient: 0.82), the geometric mean fold rise of drifted antigen-specific antibody titers was comparable with that of vaccine antigen-specific antibody titers, although the pre- and postvaccination GMTs of drifted antigen-specific antibodies were 10-fold lower than the those of the vaccine antigen-specific antibodies, in terms of microneutralization titer.^[13,15]^ As the cross-reactivities of antibodies may vary depending on the age of vaccinees and the profile of antigens, further studies are needed to understand properly the cross-reactivities of antibodies induced by IIV especially in young children.

The vaccine strain lineage of B had changed across the 4 seasons that were examined (2003/2004: Victoria lineage; 2004/2005 and 2005/2006: Yamagata lineage; 2006/2007: Victoria lineage). The SP^40^ for the Victoria lineage of the 2006/2007 vaccine component exceeded a sufficient level (70%) only in 3-year-olds who received vaccines in all 3 seasons and thus were definitively exposed to both lineages, otherwise SP^40^s were quite low even after the second dose. In contrast, the 2-year-olds only had the opportunity to receive the Yamagata lineage in both of the previous 2 seasons. Even if they had received the vaccines in the prior 2 seasons, their SP^40^ was still low after the second dose (29%) which was lower than SP^40^ in the vaccine-naïve subjects (44%). This observation may suggest that one lineage of B could not prime the immune response to the other lineage of B, which is consistent with the results of previous studies.^[8,14,16]^

The dose-response effect of the number of previously vaccinated seasons on the SP^40^s was limited. However, this result does not rule out the necessity for annual vaccination. The reasons are as follows: when we analyzed the data of the previously vaccinated 2- and 3-year-olds in detail, almost every subject received IIV in the preceding season (ie, 2005/2006 season): 47 of 48 (98%) of 2-year-olds and 58 of 61 (95%) of 3-year-olds. Moreover, the multivariate logistic model in which “vaccinated in at least 1 prior season” was changed to “vaccinated in the preceding season” led to a similar result as the original model with the former variable (data not shown). It is therefore plausible that the effect of vaccination in the preceding season was so strong that it concealed the dose-response effect of the number of previously vaccinated seasons. When we applied the same analysis to SP^160^ instead of SP^40^, the number of subjects whose antibody titer achieved 1:160 was too small to allow the dose-response effect of the number of previously vaccinated seasons on SP^160^ to be properly discussed.

With regard to AH3, we observed that experience of FRI in the preceding season significantly increased SP^160^, although we did not find such an effect on SP^40^. As mentioned before, the vaccine strain of AH3 in the study season was antigenically similar to the predominantly circulated strain in the preceding season. Provided that FRI in our data reflected influenza virus infections, this result suggests the possibility that influenza virus infections in the prior seasons may improve the immunogenicity of similar vaccine strains in children aged ≤ 3 years.

In the case of AH3, the trend of multivariate ORs that assessed the influence of age on SP was not significant in the model that included prior vaccination status as a potential confounder. However, such a trend turned out to be significant after prior vaccination status was removed from the model for SP^40^ (Supplementary Table 2). A similar tendency was also observed for SP^160^, though it was not significant (Supplementary Table 5). The results suggest that the effect of prior vaccinations on SP for AH3 was strong enough to overwhelm the effect of age.

We previously reported that younger age was a predictor of lower immunogenicity of IIV independent of baseline antibody titer level, as a result of a study of Japanese children aged 6 months to 3 years in the 2005/2006 season.^[17]^ Since the dosage of IIV used in the 2005/2006 study was below the current standard, especially for 0-year-olds (0.1 mL/dose vs 0.25 mL/dose), the possibility remains that the lower response at the younger age was due to the low dosage. This interpretation of the result had been consistent with a previous study in Japanese children.^[18]^ In contrast, in the present study using the current standard dosage, we re-confirmed the lower response in younger children when prior vaccination status was not taken into consideration, which is consistent with the results of previous studies involving the current dosage.^[3,4]^ Therefore, the low response at younger ages may be partly due to age rather than the lower dosage.

The number of withdrawals in our study was low: only one subject did not receive the second dose and serum samples of only one subject were not collected. Only one subject deviated from the protocol with regard to dosing interval. We could not rule out the possibility that guardians who were compliant with the study protocol were more likely to consent to participate in our study. However, such selection was probably independent of immunogenicity, and would therefore not have led to any bias. In our study, all study participants, including the pediatricians and guardians of subjects, were not notified of the antibody titers of the children during the serum-sampling period.

Our study is limited by 3 potential sources of information bias. First, the information on previous vaccinations was collected from the guardians using a self-administered questionnaire only, and was not confirmed using medical records or immunization registers. This bias would have resulted in underestimation of the effects of prior vaccination on immunogenicity.^[19,20]^ Second, the guardians were more likely to remember the vaccine from the preceding season than the one given 2 or 3 years ago. This bias might have impaired our ability to properly detect the dose-response effect of the number of previously vaccinated seasons on immunogenicity. Third, we used FRI in the preceding season as a surrogate of prior influenza infection. However, with respect to SP^40^s, we did not observe significant effects of prior influenza infection for all 3 strains. Using FRI might have led to some misclassification, which would have resulted in the underestimation of the effects of prior natural infection on immunogenicity. Further, we classified a subject with missing data on the experience of FRI as “the subject had no experience of FRI in the preceding season”. If this classification was incorrect, it would have contributed to underestimation of the effects of prior natural infection.

Additionally, 4 other limitations of this study should be discussed. First, the dosage of prior vaccinations was lower (0.1 mL/dose for children aged 6 months to < 1 year, 0.2 mL/dose for children aged 1 to < 6 years) than the current standard dosage. Nevertheless, prior vaccination with the lower dosage improved immunogenicity in the subsequent season. The improvement of immunogenicity using the current standard dosage was expected to be greater. Second, the difference in immunogenicity between 3-year-olds and the other ages was possibly confounded by the difference in age-specific dosage, because the dosage for 3-year-olds in the present season was double that for 0- to 2-year-olds. Third, residual confounders might also have been present. Fourth, regarding the external validity of our study results, as the immunogenicity of IIV may vary depending on the immune status of vaccines and the profile of antigens, accumulation of studies in different populations and seasons are needed to understand properly age-specific immunogenicity and the effect of prior vaccinations in young children.

## 5 Conclusion

We found that prior vaccinations improved the poor immunogenicity of IIV in young children. The previously vaccinated 1-year-olds showed immunogenicity that was comparable with those of the previously vaccinated 2- and 3-year-olds. The effect of prior vaccinations on the immunogenicity of IIV was most pronounced in the 1-year-olds. The extent of the effect of prior vaccination on improvement of the immune response may diminish in older children, probably due to increased opportunities for natural infection or vaccination.

## Author contributions

**Conceptualization:** Kazuya Ito, Wakaba Fukushima, Yoshio Hirota.

**Data curation:** Shin Irie, Motoki Ishibashi.

**Formal analysis:** Kazuya Ito, Ayumi Mugitani.

**Funding acquisition:** Yoshio Hirota.

**Investigation:** Yoshio Takasaki, Shizuo Shindo, Takashi Yokoyama, Yuji Yamashita, Keigo Shibao, Hideki Koyanagi.

**Methodology:** Kazuya Ito, Ayumi Mugitani, Wakaba Fukushima, Satoko Ohfuji, Yoshio Hirota.

**Project administration:** Shin Irie, Motoki Ishibashi.

**Resources:** Shin Irie, Wakaba Fukushima, Yoshio Hirota.

**Supervision:** Wakaba Fukushima, Yoshio Hirota.

**Validation:** Wakaba Fukushima, Satoko Ohfuji.

**Writing – original draft:** Kazuya Ito.

**Writing – review & editing:** Kazuya Ito, Wakaba Fukushima, Satoko Ohfuji, Akiko Maeda, Tetsuo Kase.

## Supplementary Material

Supplemental Digital Content
