# Supplementary material for: Prior vaccinations improve immunogenicity of inactivated influenza vaccine in young children aged 6 months to 3 years: A cohort study
Source: Medicine (Baltimore). 2018 Jul 20;97(29):e11551. doi: 10.1097/MD.0000000000011551 (PMC6086539; doi:10.1097/MD.0000000000011551)
Supplement: Supplemental Digital Content [file medi-97-e11551-s001.docx]

Supplementary Table 1. Seroprotection rate with a threshold of 1:40, according to the number of prior vaccinated seasons among 2- and 3-year-olds who had received vaccines in at least 1 previous season

|  | N |  | AH1 | | | | | |  | AH3 | | | | | |  | B | | | | | |
| --- | --- | --- | --- | --- | --- | --- | --- | --- | --- | --- | --- | --- | --- | --- | --- | --- | --- | --- | --- | --- | --- | --- |
|  |  |  | S0 | | S1 | | S2 | |  | S0 | | S1 | | S2 | |  | S0 | | S1 | | S2 | |
|  |  |  | n | (%) | n | (%) | n | (%) |  | n | (%) | n | (%) | n | (%) |  | n | (%) | n | (%) | n | (%) |
|  |  |  |  |  |  |  |  |  |  |  |  |  |  |  |  |  |  |  |  |  |  |  |
| 2-year-olds | | | | | | | | | | | | | | | | | | | | | | |
| Number of prior vaccinated seasons | | | | | | | | | | | | | | | | | | | | | | |
| 1 season | 34 |  | 3 | (9) | 29 | (85) | 32 | (94) |  | 15 | (44) | 30 | (88) | 32 | (94) |  | 0 | (0) | 10 | (29) | 10 | (29) |
| 2 seasons | 14 |  | 5 | (36) | 12 | (86) | 12 | (86) |  | 7 | (50) | 12 | (86) | 13 | (93) |  | 0 | (0) | 3 | (21) | 4 | (29) |
| p-value^#^ |  |  | .04 | | > .99 | | .57 | |  | .14 | | > .99 | | > .99 | |  | NA | | .73 | | > .99 | |
|  |  |  |  |  |  |  |  |  |  |  |  |  |  |  |  |  |  |  |  |  |  |  |
| 3-year-olds | | | | | | | | | | | | | | | | | | | | | | |
| Number of prior vaccinated seasons | | | | | | | | | | | | | | | | | | | | | | |
| 1 season | 15 |  | 5 | (33) | 15 | (100) | 15 | (100) |  | 9 | (60) | 13 | (87) | 14 | (93) |  | 0 | (0) | 6 | (40) | 6 | (40) |
| 2 seasons | 34 |  | 9 | (26) | 33 | (97) | 33 | (97) |  | 19 | (56) | 28 | (82) | 28 | (82) |  | 0 | (0) | 11 | (32) | 17 | (50) |
| 3 seasons | 12 |  | 7 | (58) | 12 | (100) | 12 | (100) |  | 9 | (75) | 12 | (100) | 12 | (100) |  | 2 | (17) | 9 | (75) | 8 | (67) |
| p-value^#^ |  |  | .22 | | > .99 | | > .99 | |  | .47 | | .41 | | .77 | |  | .04 | | .09 | | .17 | |
|  |  |  |  |  |  |  |  |  |  |  |  |  |  |  |  |  |  |  |  |  |  |  |
| N: number of subjects S1: post first vaccination titer; S2: post second vaccination titer; ^#^: Chi-squared test, Cochran-Armitage trend test or exact method; NA: not applicable due to zeros. | | | | | | | | | | | | | | | | | | | | | | |

Supplementary Table 2. Effect estimates of age on seroprotection rate with a threshold of 1:40, without adjustment of prior vaccination status

|  | Multivariate OR^†^ (95% CI) for SP^40^ | | | | | | | | | | | | | |  |
| --- | --- | --- | --- | --- | --- | --- | --- | --- | --- | --- | --- | --- | --- | --- | --- |
|  | AH1 | | | |  | AH3 | | | |  | B | | | | |
|  | S1 | | S2 | |  | S1 | | S2 | |  | S1 | | S2 | | |
|  |  | |  | |  |  | |  | |  |  | |  | | |
| Age | | | | | | | | | | | | | | |  |
| 1-year-olds | 1.0 |  | 1.0 |  |  | 1.0 |  | 1.0 |  |  | 1.0 |  | 1.0 |  | |
| 2-year-olds | 4.5 | (2.1-10) | 2.4 | (0.9-7.3) |  | 2.3 | (1.0-5.2) | 6.7 | (2.7-19) |  | 3.7 | (1.2-13) | 0.6 | (0.3-1.3) | |
| 3-year-olds | 20 | (6.4-88) | 15 | (2.7-285) |  | 3.8 | (1.6-9.6) | 3.1 | (1.3-7.9) |  | 7.6 | (2.7-25) | 1.2 | (0.6-2.4) | |
| P trend^‡^ | < .01 | | < .01 | |  | < .01 | | < .01 | |  | < .01 | | .73 | | |
|  |  |  |  |  |  |  |  |  |  |  |  |  |  |  | |
| Prevaccination HI antibody titer | | | | | | | | | | | | | | |  |
| < 1:10 | 1.0 |  | 1.0 |  |  | 1.0 |  | 1.0 |  |  | 1.0 |  | 1.0 |  | |
| 1:10 - 1:20 | 2.5 | (1.2-5.5) | 1.5 | (0.5-3.8) |  | 5.3 | (1.7-20) | 1.5 | (0.5-4.1) |  | 12.1 | (4.2-41) | 12.1 | (3.8-54) | |
| ≥1:40 | 12 | (2.1-237) | 2.7 | (0.4-55) |  | 55 | (14-272) | 30 | (6.6-221) | | NA | | NA | | |
| P trend^‡^ | < .01 | | .31 | |  | < .01 | | < .01 | |  | < .01 | | < .01 | | |
|  |  |  |  |  |  |  |  |  |  |  |  |  |  |  | |
| Febrile respiratory illness in the preceding season | | | | | | | | | | | | | | |  |
| No | 1.0 |  | 1.0 |  |  | 1.0 |  | 1.0 |  |  | 1.0 |  | 1.0 |  | |
| Yes | 1.4 | (0.7-3.0) | 0.5 | (0.2-1.1) |  | 1.0 | (0.5-2.0) | 1.4 | (0.7-3.0) |  | 2.1 | (0.98-4.6) | 1.1 | (0.6-1.9) | |
|  |  |  |  |  |  |  |  |  |  |  |  |  |  |  | |
| OR: odds ratio; CI: confidence interval; S1: post vaccination titer after the first dose; S2: post vaccination titer after the second dose; NA: not applicable due to the small number of subjects whose prevaccination titer was ≥1:40 (no subject in 1- to 2-year-old group and 2 subjects in 3-year-old group).  †: Model included all variables shown in the table; ‡: Wald chi-square test for the model parameter. | | | | | | | | | | | | | | |  |

Supplementary Table 3. Seroprotection rate with a threshold of 1:160 according to prior vaccination within each age stratum

|  | N |  | AH1 | | | | | |  | AH3 | | | | | |  | B | | | | | |
| --- | --- | --- | --- | --- | --- | --- | --- | --- | --- | --- | --- | --- | --- | --- | --- | --- | --- | --- | --- | --- | --- | --- |
|  |  |  | S0 | | S1 | | S2 | |  | S0 | | S1 | | S2 | |  | S0 | | S1 | | S2 | |
|  |  |  | n | (%) | n | (%) | n | (%) |  | n | (%) | n | (%) | n | (%) |  | n | (%) | n | (%) | n | (%) |
|  |  |  |  |  |  |  |  |  |  |  |  |  |  |  |  |  |  |  |  |  |  |  |
| 0-year-olds | 55 |  | 1 | (2) | 1 | (2) | 13 | (24) |  | 3 | (5) | 3 | (5) | 4 | (7) |  | 0 | (0) | 0 | (0) | 2 | (4) |
|  |  |  |  |  |  |  |  |  |  |  |  |  |  |  |  |  |  |  |  |  |  |  |
| 1-year-olds | | | | | | | | | | | | | | | | | | | | | | |
| Vaccination in the preceding season | | | | | | | | | | | | | | | | | | | | | | |
| Never | 54 |  | 0 | (0) | 2 | (4) | 11 | (20) |  | 5 | (9) | 5 | (9) | 6 | (11) |  | 0 | (0) | 0 | (0) | 1 | (2) |
| Vaccinated | 22 |  | 0 | (0) | 9 | (41) | 6 | (27) |  | 4 | (18) | 12 | (55) | 10 | (45) |  | 0 | (0) | 0 | (0) | 0 | (0) |
| p-value^#^ |  |  | NA | | < .01 | | .55 | |  | .43 | | < .01 | | < .01 | |  | NA | | NA | | > .99 | |
|  |  |  |  |  |  |  |  |  |  |  |  |  |  |  |  |  |  |  |  |  |  |  |
| 2-year-olds | | | | | | | | | | | | | | | | | | | | | | |
| Vaccination during the prior 2 seasons | | | | | | | | | | | | | | | | | | | | | | |
| Never | 18 |  | 2 | (11) | 3 | (17) | 12 | (67) |  | 2 | (11) | 3 | (17) | 3 | (17) |  | 0 | (0) | 1 | (6) | 3 | (17) |
| Vaccinated^*^ | 48 |  | 2 | (4) | 17 | (35) | 15 | (31) |  | 10 | (21) | 25 | (52) | 27 | (56) |  | 0 | (0) | 3 | (6) | 4 | (8) |
| p-value^#^ |  |  | .30 | | .13 | | .01 | |  | .49 | | .01 | | < .01 | |  | NA | | > .99 | | .38 | |
|  |  |  |  |  |  |  |  |  |  |  |  |  |  |  |  |  |  |  |  |  |  |  |
| 3-year-olds | | | | | | | | | | | | | | | | | | | | | | |
| Vaccination during the prior 3 seasons | | | | | | | | | | | | | | | | | | | | | | |
| Never | 8 |  | 1 | (13) | 1 | (13) | 3 | (38) |  | 4 | (50) | 4 | (50) | 4 | (50) |  | 0 | (0) | 0 | (0) | 1 | (13) |
| Vaccinated^*^ | 61 |  | 2 | (3) | 35 | (57) | 32 | (52) |  | 26 | (43) | 36 | (59) | 35 | (57) |  | 0 | (0) | 11 | (18) | 13 | (21) |
| p-value^#^ |  |  | .31 | | .02 | | .48 | |  | .72 | | .71 | | .72 | |  | NA | | .34 | | > .99 | |
|  |  |  |  |  |  |  |  |  |  |  |  |  |  |  |  |  |  |  |  |  |  |  |
| N: number of subjects; S0: baseline titer; S1: post vaccination titer after the first dose; S2: post vaccination titer after the second dose; NA: not applicable due to zeros.  *: Subjects were categorized as “Vaccinated”, if they had received IIV in at least 1 of the prior seasons; #: Chi-squared test or Fisher’s exact method. | | | | | | | | | | | | | | | | | | | | | | |

Supplementary Table 4. Effect estimates of prior vaccination on seroprotection rate with a threshold of 1:160

|  | Multivariate OR^†^ (95% CI) for SP^160^ | | | | | | | | | | | | | |
| --- | --- | --- | --- | --- | --- | --- | --- | --- | --- | --- | --- | --- | --- | --- |
|  | AH1 | | | |  | AH3 | | | |  | B | | | |
|  | S1 | | S2 | |  | S1 | | S2 | |  | S1 | | S2 | |
|  |  | |  | |  |  | |  | |  |  | |  | |
| Vaccination during the prior 3 seasons | | | | | | | | | | | | | | |
| Never | 1.0 |  | 1.0 |  |  | 1.0 |  | 1.0 |  |  | 1.0 |  | 1.0 |  |
| Vaccinated^*^ | 7.1 | (2.7-22) | 0.6 | (0.3-1.3) |  | 19 | (5.6-73) | 8.2 | (3.0-25) |  | 4.5 | (0.6-94) | 0.7 | (0.2-2.9) |
|  |  |  |  |  |  |  |  |  |  |  |  |  |  |  |
| Age | | | | | | | | | | | | | | |
| 1-year-olds | 1.0 |  | 1.0 |  |  | 1.0 |  | 1.0 |  |  | NA | | 1.0 |  |
| 2-year-olds | 0.7 | (0.2-2.0) | 2.3 | (1.0-5.5) |  | 0.4 | (0.1-1.3) | 0.9 | (0.3-2.6) |  | NA | | 6.9 | (1.0-141) |
| 3-year-olds | 1.3 | (0.5-3.6) | 3.1 | (1.3-7.7) |  | 0.4 | (0.1-1.2) | 0.6 | (0.2-1.9) |  | NA | | 12 | (1.7-250) |
| P trend^‡^ | .39 | | .02 | |  | .11 | | .39 | |  | .04 | | .02 | |
|  |  |  |  |  |  |  |  |  |  |  |  |  |  |  |
| Prevaccination HI antibody titer | | | | | | | | | | | | | | |
| < 1:10 | 1.0 |  | 1.0 |  |  | NA | | 1.0 |  |  | 1.0 |  | 1.0 |  |
| 1:10 - 1:20 | 4.0 | (1.4-15) | 1.4 | (0.6-3.1) |  | NA | | 2.9 | (0.5-55) |  | 13 | (3.6-48) | 14 | (4.6-44) |
| ≥1:40 | 15 | (4.0-66) | 5.9 | (2.1-18) |  | NA | | 97 | (16-1932) |  | 11 | (0.4-319) | NA | |
| P trend^‡^ | < .01 | | < .01 | |  | < .01 | | < .01 | |  | < .01 | | < .01 | |
|  |  |  |  |  |  |  |  |  |  |  |  |  |  |  |
| Febrile respiratory illness in the preceding season | | | | | | | | | | | | | | |
| No | 1.0 |  | 1.0 |  |  | 1.0 |  | 1.0 |  |  | 1.0 |  | 1.0 |  |
| Yes | 1.3 | (0.7-2.6) | 0.9 | (0.5-1.6) |  | 2.7 | (1.1-7.1) | 2.8 | (1.2-6.6) |  | 1.7 | (0.5-6.6) | 1.4 | (0.5-4.3) |
|  |  |  |  |  |  |  |  |  |  |  |  |  |  |  |
| OR: odds ratio; CI: confidence interval; S1: post vaccination titer after the first dose; S2: post vaccination titer after the second dose; NA: not applicable due to the small number of subjects at least one of categories.  *: Subjects were categorized as “Vaccinated”, if they had received IIV in at least 1 of the prior seasons; †: Model included all variables shown in the table; ‡: Wald chi-square test for the model parameter. | | | | | | | | | | | | | | |

Supplementary Table 5. Effect estimates of age on seroprotection rate with a threshold of 1:160, without adjustment of prior vaccination status

|  | Multivariate OR^†^ (95% CI) for SP^160^ | | | | | | | | | | | | | |
| --- | --- | --- | --- | --- | --- | --- | --- | --- | --- | --- | --- | --- | --- | --- |
|  | AH1 | | | |  | AH3 | | | |  | B | | | |
|  | S1 | | S2 | |  | S1 | | S2 | |  | S1 | | S2 | |
|  |  | |  | |  |  | |  | |  |  | |  | |
| Age | | | | | | | | | | | | | | |
| 1-year-olds | 1.0 |  | 1.0 |  |  | 1.0 |  | 1.0 |  |  | NA | | 1.0 |  |
| 2-year-olds | 1.6 | (0.7-3.9) | 1.9 | (0.9-4.1) |  | 1.2 | (0.4-3.3) | 1.9 | (0.7-5.0) |  | NA | | 6.0 | (0.9-118) |
| 3-year-olds | 3.5 | (1.5-8.4) | 2.3 | (1.1-5.1) |  | 1.6 | (0.6-4.8) | 1.9 | (0.7-5.1) |  | NA | | 10 | (1.7-192) |
| P trend^‡^ | < .01 | | .04 | |  | .36 | | .20 | |  | .01 | | .02 | |
|  |  |  |  |  |  |  |  |  |  |  |  |  |  |  |
| Prevaccination HI antibody titer | | | | | | | | | | | | | | |
| < 1:10 | 1.0 |  | 1.0 |  |  | NA | | 1.0 |  |  | 1.0 |  | 1.0 |  |
| 1:10 - 1:20 | 4.9 | (1.8-17) | 1.2 | (0.6-2.7) |  | NA | | 3.1 | (0.6-59) |  | 11 | (3.3-41) | 14 | (4.6-43) |
| ≥1:40 | 16 | (4.8-68) | 5.2 | (1.9-15) |  | NA | | 76 | (14-1430) | | 12 | (0.4-346) | NA | |
| P trend^‡^ | < .01 | | < .01 | |  | < .01 | | < .01 | |  | < .01 | | < .01 | |
|  |  |  |  |  |  |  |  |  |  |  |  |  |  |  |
| Febrile respiratory illness in the preceding season | | | | | | | | | | | | | | |
| No | 1.0 |  | 1.0 |  |  | 1.0 |  | 1.0 |  |  | 1.0 |  | 1.0 |  |
| Yes | 1.4 | (0.7-2.7) | 0.9 | (0.5-1.6) |  | 2.5 | (1.1-6.2) | 2.7 | (1.2-6.1) |  | 1.5 | (0.4-5.3) | 1.4 | (0.5-4.4) |
|  |  |  |  |  |  |  |  |  |  |  |  |  |  |  |
| OR: odds ratio; CI: confidence interval; S1: post vaccination titer after the first dose; S2: post vaccination titer after the second dose; NA: not applicable due to the small number of subjects at least one of categories.  †: Model included all variables shown in the table; ‡: Wald chi-square test for the model parameter. | | | | | | | | | | | | | | |
